# Association of *Helicobacter pylori* infection with coronary artery disease: is it an independent risk factor?

**DOI:** 10.1186/s43044-021-00185-2

**Published:** 2021-07-03

**Authors:** Mohamed Riad

**Affiliations:** Mansoura University Faculty of Medicine, Elgomhouria street, Mansoura city, Dakahlia Egypt

**Keywords:** *Helicobacter pylori*, Coronary artery disease, Infection, Atherosclerosis, Dyslipidemia, Thrombosis, Stroke

## Abstract

**Background:**

Coronary artery disease (CAD) is a dilemma and a serious cause of morbidity and mortality worldwide. Since CAD has been seen in people without the conventional risk factors like smoking, diabetes, and hypertension, the infectious theory being a risk factor has arisen.

**Main body:**

*Helicobacter pylori* (HP) infection is the most common infection affecting the vast majority of the population worldwide. HP grows in the gastrointestinal tract (GIT) and responsible for chronic gastritis, peptic ulcer, gastric adenocarcinoma, and gastric lymphoma. A review of medical literature mainly PubMed has revealed several studies reporting that HP pathogenesis extends beyond the GIT to be a predisposing factor for atherosclerosis, dyslipidemia, thrombosis, and CAD. However, it remains a controversial issue that warrants extensive research.

**Conclusion:**

This article gives insight into the diversity of opinions, evidence, and theories regarding the association between HP infection and CAD. The idea that CAD may be managed with antibiotics in certain patients seems to be creative and inspiring. More research is mandatory to either verify or reject this proposed correlation with strong scientific evidence and also to demonstrate the implications of the results on CAD management and outcome.

## Background

Coronary artery disease, also known as ischemic heart disease or atherosclerotic heart disease, is a narrowing of the coronary arteries which supply the heart with oxygen and nutrients. CAD is usually the end result after decades of atherosclerosis and atherosclerotic plaque accumulation limiting the blood flow to the heart. This leads to variable clinical presentations ranging from stable angina, unstable angina, and myocardial infarction (MI) to even sudden cardiac death. Because the atherosclerotic process is gradual and slow occurring over decades of life, the majority of patients with CAD show no signs or symptoms until the most advanced stage of the disease. Unfortunately, some CAD patients may be asymptomatic for years and firstly present with a sudden attack of MI. Another shocking fact is that the first presentation of CAD can be sudden cardiac death. About 75% of sudden cardiac deaths are due to CAD and many cases of sudden cardiac deaths occur in asymptomatic CAD patients who are not aware of having the disease and the risk varies among those patients according to the presence or absence of other risk factors such as hypercholesterolemia, hypertension, obesity, diabetes, smoking, and cardiac abnormalities [1].

In general, CAD is the leading cause of morbidity and mortality all over the world accounting for about seven million deaths per year, and one death due to MI occurs each minute in the USA [2, 3]. CAD is not a disease of older adult men only. People of any age, gender, and racial or ethnic group can develop CAD, so no one is immunized against it. CAD is still a major serious public health and clinical issue in spite of the recent development in the diagnostic and therapeutic modalities. That may be because we have not covered all aspects of this serious disease and there is still much to discover and know about it. The most commonly established risk factors of CAD include modifiable and non-modifiable causes. The non-modifiable risk factors are age, gender, and genetic predisposition. The modifiable causes include atherosclerosis, hyperlipidemia, obesity, diabetes, hypertension, smoking, chronic kidney disease, metabolic syndrome, and homocystinuria. In addition to the previously mentioned risk factors, chronic bacterial or viral infections have been reported to be linked to the pathogenesis of atherosclerosis and CAD [4]. Interestingly, some studies have discovered an association between *Helicobacter pylori* (HP) infection and CAD.

HP is the most frequent and highest prevalent infection worldwide, which is caused by gram-negative acidophilic spiral-shaped bacteria, and primarily inhabits the stomach and duodenum. It is estimated that more than half of the world’s population and about 40% of the US population are having HP infection. The incidence is higher in developing countries. Unsanitary food and water, bad hygiene, lower socio-economic status, overcrowding, smoking, and dealing with someone already infected increase the risk of having HP infection. Discovery and understanding of HP infection have had several important implications in clinical medicine. Recently, HP has been recognized as an infectious disease that requires the medical attention whether the affected people have signs or symptoms or not [5]. HP infection has been identified as the main cause of acute gastritis, chronic atrophic gastritis, peptic ulcer, gastric adenocarcinoma, and MALT (mucosa-associated lymphoid tissue) lymphoma. However, the pathology of HP is not confined to the gastrointestinal tract. HP is found to be associated with other extra-gastric diseases such as idiopathic iron deficiency anemia, vitamin B12 deficiency, immune thrombocytopenic purpura (ITP) and other hematologic disorders, neurodegenerative diseases like Alzheimer’s disease and Parkinson disease, and an increased risk of preeclampsia in the infected women [6–9].

In addition, HP has been recently reported as a culprit of atherosclerosis and the inflammatory process resulting in CAD and cerebrovascular disease (CVD) [10]. The aim of this review article is to shed the light on the relationship between chronic HP infection and the progression of atherosclerosis ending up with the development of CAD as well as the impact of HP infection on cardiovascular morbidity and mortality. This review will critically discuss the pathologic mechanism by which chronic HP infection contributes to the occurrence of atherosclerosis, ischemia, and CAD. It will also address the variations, contradictions, and limitations in the data that is talking about this unusual association.

## Main text

### The association between HP infection and atherosclerosis

Atherosclerosis is a serious healthcare issue that is precedent to other major health problems like CAD, CVD, peripheral vascular disease (PVD), or even death. It is considered a chronic inflammatory process of the arterial wall including two components: abnormal lipid metabolism and abnormal immune reaction. Atherosclerosis is a multifactorial process that is usually initiated at a young age and remains sub-clinical for many years before the clinical manifestations develop. Hypertension, hyperlipidemia, smoking, and diabetes mellitus are common established causes of atherosclerosis. Apart from that, it has been confirmed that atherosclerosis has an infectious etiology, and certain infections are triggering and promoting the atherosclerotic process. These infectious agents include *H. pylori*, chlamydia pneumonia, Epstein-Barr virus (EBV), and cytomegalovirus (CMV) [11, 12].

The atherosclerosis process develops through the following main events:

- Endothelial damage: This is the initial step in atherosclerotic plaque formation.
- Lipoprotein (LDL) and cholesterol deposition into the damaged arterial wall: After endothelium disruption, LDL molecules enter the vessel wall, get oxidized by free radicals, then get engulfed by macrophages forming foam cells and fatty streaks.
- Inflammatory reaction: The oxidized LDL and the injured endothelium release several inflammatory mediators or cytokines leading to the recruitment of other inflammatory cells like macrophages into the vessel wall.
- Smooth muscle cells and fibrous cap formation: smooth muscle proliferation and migration from tunica media to intima in response to the cytokines secreted by damaged endothelial cells. This results in the formation of a fibrous capsule covering the fatty streak.
- Plaque necrosis and rupture: the atherosclerotic plaque is an accumulation of cholesterol, white blood cells, calcium, and other substances in the walls of arteries. Eventually, it leads to narrowing of the lumen of arteries and stiffening of the wall. Stable plaques are less likely to rupture. Unstable plaques, which undergo thinning of the fibrous capsule, are more prone to rupture with thrombus formation on top of the ruptured plaque. Inflammation within the plaque makes the fibrous cap unstable and increases the likelihood of rupture.

Cytotoxin-associated gene-A (CagA) is an important virulence factor that has been studied to be involved in the atherosclerotic process. In a systematic meta-analysis of 26 case-control studies, 11 studies of them were on ischemic stroke and 15 studies were on coronary heart disease. It has concluded that the infection with CagA-seropositive HP strains is predisposing to CVD and CAD [13]. Another study was conducted to analyze five issues:

- To determine the seroprevalence of *H. pylori* and its CagA in patients with and without CAD (group A).
- To evaluate the influence of infection with H. pylori expressing CagA on coronary arterial lumen reduction in patients after percutaneous transluminal coronary angioplasty (PTCA) with stent (group B).
- To assess the effect of H. pylori eradication on coronary artery lumen reduction after PTCA (group C).
- To determine the influence of the *H. pylori* eradication on plasma levels of cytokines, lipids, and coagulation factors in the same patients before and after PTCA (group C).
- To analyze coronary specimens in patients with severe CAD for the presence of H. pylori originated specific DNA (Group D).

This study has established that there is a significant correlation between atherosclerosis causing CAD and HP infection particularly CagA-positive strains. HP infection increases the risk of restenosis after PTCA. Eradication of HP was associated with a decreased level of cytokines and inflammatory mediators suggesting HP main role in inducing inflammation. Moreover, HP DNA was isolated from the atherosclerotic plaques of patients with severe CAD confirming that association [14].

The proposed mechanisms by which CagA-positive HP strains cause atherosclerosis include increasing COX-1 and COX-2 production from the endothelium of blood vessels leading to increasing the synthesis of prostaglandins and thromboxane A2 (TXA2) which induce platelets’ aggregation. In addition, these HP strains can trigger a strong inflammatory reaction by releasing a huge amount of cytokines such as interleukin-1 (IL-1), interleukin-1 (IL-6), and tumor necrosis factor-α (TNF-α) free radicals causing oxidative stress and atherosclerosis. C-reactive protein (CRP) is an important inflammatory marker that is frequently elevated in chronic HP infection. Furthermore, an abnormal immune reaction is thought to be involved in atherosclerotic plaque destabilization and rupture through the cross-reactivity between anti-CagA Antibodies and vascular wall antigens [15–19].

Since inflammation and its associated pro-inflammatory cytokines and immune cells are representing a major event in the process of atherosclerosis, other studies support the immune cross-reactivity mechanism by reporting the finding of an antigenic protein in HP which is similar to a protein called heat shock protein-60 present in endothelial cells resulting in abnormal autoimmune reaction, releasing different cytokines and inflammation of the vascular wall [20–23]. In addition, other studies emphasize the previously mentioned idea of the presence of HP DNA in the atherosclerotic plaque acting like a nidus of infection and inflammation [24–27].

### The correlation between HP infection and dyslipidemia

Dyslipidemia means high levels of low-density lipoproteins (LDL) and triglycerides (TGs) or a low level of high-density lipoproteins (HDL), predisposing to atherosclerosis [28]. A study done on 961 patients concluded that HP is not associated directly with the severity of CAD; however, it is associated with low HDL level which has a protective role against atherosclerosis [29].

Dyslipidemia is one of the possible mechanisms by which HP infection may lead to CAD. In patients with HP infection, derangements in their lipid profile were found in the form of low HDL cholesterol and high total cholesterol, LDL cholesterol, and triglyceride levels [30, 31] Therefore, it is necessary to continuously monitor the lipid profile in those patients [32].

### The correlation between HP infection and thrombosis

Arterial thrombosis has been reported as a consequence of chronic HP infection through various proposed mechanisms. Chronic HP infection is associated with micronutrient deficiency especially vitamin B12 and folate. The exact mechanism underlying this deficiency is not fully understood [33–35]. Hyperhomocysteinemia arises as a consequence of vitamin B12 or folate deficiency leading to endothelial injury, thrombosis, and increasing the risk of CAD even premature CAD [36, 37]. Moreover, CagA-positive HP strains increase COX-1 and COX-2 production resulting in increasing TXA2 which stimulates platelet aggregation [15, 16]. It also causes stimulation of the coagulation pathway. Additionally, the intense inflammatory condition associated with HP infection results in high circulatory amounts of cytokines (IL-1, IL-6, and TNF-α) and pro-coagulant pro-thrombotic proteins such as thrombin, fibrinogen, and some adhesion molecules like intercellular adhesion molecule-1 (ICAM-1). In general, chronic HP infection is associated with a state of sustained inflammation and hypercoagulability making the seropositive patients vulnerable to thrombosis formation. Thrombosis may affect the coronary circulation, cerebral circulation, or peripheral arteries.

### The association between HP infection and CAD and myocardial infarction

Myocardial infarction (MI) is the most serious and most feared outcome in CAD patients because it can be fatal or affect the quality of life afterward in the survivors. As a result of the chronic inflammatory and hypercoagulability state induced by HP infection, it may be a culprit of the acute coronary syndrome (ACS) and (MI). The proposed theories of HP infection-related CAD are summarized in Table 1. Numerous studies have demonstrated the link between HP infection and CAD. A meta-analysis of 26 studies involving more than 20,000 participants found that HP infection increases the risk of MI even in young people [38]. Another large case-control study detected a high prevalence of HP infection (42% vs. 24%, OR = 1.75) in young patients after early-onset acute myocardial infarction (AMI) in 1122 survivors; in addition to finding that was MI was twice as common in individuals seropositive for HP as in those seronegative [39]. In a study measuring HP antibodies titer and comparing it to the outcome of CAD, a higher HP antibodies (IgG) titer was detected in patients who died of AMI (151 ng/mL vs. 88 ng/mL, p= 0.034) [40]. Another prospective study was investigating the correlation between HP infection and CAD including the myocardial perfusion imaging (MPI) stress test. It has confirmed the association between HP infection and CAD and MI independent of the conventional risk factors [41]. Several studies have demonstrated the correlation between HP infection and premature CAD in absence of the traditional cardiovascular risk factors [42, 43]. A systematic review and meta-analysis of 44 studies involving 7522 cases and 8311 controls concluded that HP infection increases the risk of ACS (OR = 2.03, 95% CI 1.66–2.47) [44].

**Table 1:** Theories of the association between HP infection and CAD

Theories suggesting the association between HP infection and CAD	
Cross-reactivity	
Sustained chronic inflammation with subsequent oxidative stress and cytokines release	
Dyslipidemia	
Induction of a hypercoagulable state	
Hyperhomocysteinemia	

On the other hand, the association between HP infection and CAD is still debatable and controversial. At the same time, other studies are demonstrating the lack of association between HP infection and CAD. A study performed on 112 patients who were the candidate of coronary angiography concluded that there is no significant association between CagA-positive HP and the severity of CHD; however, this result may be due to low sample size and if it is done on a larger number of patients, it may yield different results [45]. Another large prospective study ruled out the association between HP infection and CAD mortality [46]. A meta-analysis of five prospective studies concluded that there is no significant correlation between H. pylori infection with CAD (RR = 1.13) [47]. Regarding the risk of restenosis following PTCA, a study involving 180 patients who had stent implantation in a native coronary artery with systematic angiographic and intravascular ultrasound (IVUS) follow-up at 6 months reported no association between HP infection and restenosis after PTCA [48].

### The correlation between HP infection and other vascular diseases

Some studies are discussing HP infection as a risk factor to ischemic stroke and PVD as well and the impact of HP eradication on those vascular disorders; however, there are limited data and few studies about those associations.

At the end of this discussion, the relationship between HP infection and CAD has been controversial over the past years and there is no strong scientific evidence establishing this association. Supporters and opponents are confirming and ruling out the role of HP infection in CAD, so well-organized, high-quality, evidence-based, and less biased studies in the form of cross-sectional, cohort, and randomized clinical trials are necessary to assess and get to a definitive conclusion regarding this debatable correlation. Moreover, if it is confirmed to be a significant correlation, will HP eradication prevent the development of CAD and its complications? So large prospective studies comparing HP eradication in individuals who are at risk or already have CAD and then follow up to assess the risk or outcome of CAD may help us reach conclusive evidence.

## Conclusion

HP infection and CAD have used to be a controversial link and a mystery that has baffled many researchers for years. The available data suggested that HP infection can be considered a risk factor for CAD. Meanwhile, other data reported that this association is coincidental. Further large, comprehensive, and high-quality studies are required to investigate this association thoroughly and reach a definitive decision based on the previously discussed considerations and extend beyond that as well. In case of being proved as significant correlation, the eradication of this infection followed by its reassessment in patients after eradication can clarify the role of this organism in such pathogeneses. This will help to save the lives of many CAD patients and improve their quality of life by just managing HP infection, in addition to its strong positive socioeconomic impact all over the world.

## Data Availability

Reviewing of medical literature using PubMed, Google Scholar, and some other gray literature from the World Health Organization (WHO) and Centers for Disease Control and Prevention (CDC). All data and materials of the article are available and accessible.
